# Risk Attitude in Multicriteria Decision Analysis: A Compromise Approach

**DOI:** 10.3390/ijerph18126536

**Published:** 2021-06-17

**Authors:** Juan Ribes, Jacinto González-Pachón

**Affiliations:** 1 ETSI Informáticos, Campus de Montegancedo, Universidad Politécnica de Madrid, Boadilla del Monte, 28660 Madrid, Spain; 2 Department of Financial and Actuarial Economics and Statistics, Facultad de Ciencias Económicas y Empresariales, Campus de Somosaguas, Universidad Complutense de Madrid, Pozuelo de Alarcón, 28223 Madrid, Spain; 3 Department of Artificial Intelligence, Campus de Montegancedo, Universidad Politécnica de Madrid, Boadilla del Monte, 28660 Madrid, Spain

**Keywords:** stochastic multicriteria decision, stochastic efficiency, risk attitudes, Extended Goal Programming

## Abstract

In fields on which decisions need to be taken including health, as we are seeing nowadays in the COVID-19 crisis, decision-makers face multiple criteria and results with a random component. In stochastic multicriteria decision-making models, the risk attitude of the decision maker is a relevant factor. Traditionally, the shape of a utility function is the only element that represents the decision maker’s risk attitude. The eduction process of multi-attribute utility functions implies some operational drawbacks, and it is not always easy. In this paper, we propose a new element with which the decision maker’s risk attitude can be implemented: the selection of the stochastic efficiency concept to be used during a decision analysis. We suggest representing the risk attitude as a conflict between two poles: risk neutral attitude, associated with best expectations, and risk aversion attitude, associated with a lower uncertainty. The Extended Goal Programming formulation has inspired the parameter that is introduced in a new risk attitude formulation. This parameter reflects the trade-off between the two *classical* poles *with respect to risk attitude*. Thus, we have produced a new stochastic efficiency concept that we call *Compromise Efficiency*.

## 1. Introduction

The current COVID-19 crisis has shown that relevant decisions often have to be taken under uncertainty or risk environments. Moreover, these decisions need to consider several criteria during their structuring. We have seen how preventive actions, such as population confinement, which have proven to be effective for curbing the spread of the virus, also lead to other health, economic, political, ethical and social problems as side effects, see [1,2,3,4,5], among others.

Considering only the health factors, slowing down the propagation of COVID-19 by confinement has caused cases of anxiety, asthenia and depression when mental health is focused on [6]; or problems due to the lack of, or decrease in, physical activity if we focus on pediatrics [7]. The list of pathologies in these and other disciplines is longer and may be a subject for study in Medicine for many years.

Would it have been better not to have proceeded to a massive confinement so as not to produce those side effects? We will never know. Certainly letting the virus run loose could have quickly caused many deaths and a collapse of the hospital system and of corpse handling facilities. It is easy to predict that this would have led to other side effects on population health.

In taking decisions containing preventive measures, different national authorities have faced different criteria, whose outcome, once an alternative had been chosen, was not fully predictable. And was uncertain, or at least had a random component. As an example, no one could tell ahead of time how long a confinement period would be needed to decelerate the propagation of COVID-19, and we are still unable to evaluate all its side effects. But decisions had to be taken before the effects were confirmed.

This is an usual decision-making situation: The Decision Maker (DM) needs to address simultaneously several criteria, whose outcome is uncertain. Decisions reached will depend on the DM’s attitude with respect to risk. To guide the decision process, the analyst needs to consider that risk attitude.

Under this scenario, the DM may prefer the alternative with the best expected consequences, rejecting any further analysis with respect to discrepancies in the consequences when the final scenario (state of the nature) shows up. The DMs have a risk neutral attitude; or, on the contrary, they may prefer an alternative for which the outcome is less uncertain, i.e., one for which the result is predictable when the DM has a risk averse attitude.

The analyst faces the technical challenge of reflecting those DM preferences, attitudes towards risk, in the decision model. Those attitudes could be different for each criterion, and different for each DM.

One way of measuring risk [8] is by evaluating the variance, or the standard deviation, of the underlying random variables associated with different criteria. In contrast to risk neutral attitude, in which the DM aims to optimize the expected value, a DM with a risk averse attitude will prefer to reduce the variability of consequences related to the uncertainty of the final scenario; i.e., the DM would like to be sure about the consequences of the decision taken; i.e., a risk averse DM prefers the alternative which leads to the lowest variance of the results. The expected values may be worse, but the outcome is more certain. The expected values are not considered, only the variances.

DMs with a risk moderate attitude are positioned between two poles: risk neutrality and extreme risk aversion. Therefore, the analyst needs to consider both the expected values and the variances, or standard deviations, of criteria as operational tools; and a model to address the conflict between those two poles.

Risk aversion is widely studied in Utility Theory [9], or in other paradigms such as Prospect Theory [10]. But the study of attitude in stochastic decision making is not limited to this aspect. Other psychological aspects that are relevant in decision-making have been considered, like prudence and temperance [11] or optimism and pessimism [12].

Hurwicz [12] proposed a parameter to reflect the DM’s position between optimism and pessimism. González Pachón and Romero [13,14], used Extended Goal Programming to develop the idea of a compromise consensus on majority-minority principles within a social choice environment. Inspired in both developments, here the idea is to apply a parameter, or a set of parameters to risk attitude, which allows this feature to be reflected similarly.

In this paper, we propose a method for seeking for solutions for stochastic multicriteria decision problems by taking into consideration this conflict between poles in risk aversion attitudes. And to do so by introducing a parameter, or set of them, whose values are easy to determine in the interaction between an analyst and a risk moderate DM.

This method is based on different stochastic efficiency concepts [15], their relationships in terms of efficient sets [16], and the inherent idea of Extended Goal Programming [17].

There are other approaches to the problem of reflecting the degree of risk aversion on decision problems. In [18] Zhou and Chen propose dealing with social multicriteria discrete optimization problems on a fuzzy environment using the VIKOR technique, based on compromise programming, being risk aversion of DM reflected by an utility function. In [19] Fu et al. apply multicriteria decision to the public health environment on the assessment of the benefits and risk of medical products, again reflecting patient preferences by utility functions.

Considering pure Stochastic Multicriteria Optimization, Liu and Vicente [20], in Artificial Intelligence, propose to solve the Expected value Efficiency case (see Section 2) using the concept of stochastic multi-gradient method. Nevertheless this is limited to risk neutral DMs. Also Nabavi et al. [21] deal with Stochastic Multicriteria Optimization, but, opposite to us, in the case that what is random is the restrictions.

Finally, and back to Decision Analysis, Ben Abdelaziz et al. [22] propose dealing with multicriteria stochastic decisions considering stochastic processes, where development and sustainability on time are key factors. Again, utility functions are involved on the model.

Different from approaches on Decision Analysis described above, on the method we now introduce there is no need to elicitate a utility function based on DM preferences, but only to determine parameters on risk aversion facing each criterion separately.

The structure of the paper is as follows: Section 2 reviews some basic results on the Stochastic Multicriteria Optimization Problem, including the link between efficiency concepts and DM attitudes towards risk, and relationships between associated efficient sets. Section 3 presents the *Compromise Efficiency* as a new concept for dealing with risk attitude in a stochastic multicriteria decision problem in a parametric way. Also we consider some methods to find specific solutions. Finally, we describe an illustrative case study where we solve the proposed multi-criteria Compromise Efficiency program using methods considered in Section 3.

## 2. Risk Attitudes Based on Stochastic Efficiency

Without any loss of generality, a Stochastic Multicriteria Optimization Problem with deterministic constraints (SMOP) can be defined as follows [16]:

(1) $$\begin{array}{cc} opt_{x\in F} & \tilde{z}\left(x,\tilde{\xi }\right)=\left({\tilde{z}}_{1}\left(x,\tilde{\xi }\right),\cdots ,{\tilde{z}}_{m}\left(x,\tilde{\xi }\right)\right), \end{array}$$

where each criterion ${\tilde{z}}_{k}\left(x,\tilde{\xi }\right)$ depends on a random vector $\tilde{\xi }$, *x* is the vector of decision variables and $F\subset R^{n}$ is a non empty feasible set.

Some of the methods developed for solving the general SMOP are included in what has been called the multi-objective approach, see Stancu-Minasian and Tigan [23], Ben Abdelaziz [15] or Gutjahrand and Pichler [8]. This approach consists of the transformation of the SMOP into a deterministic multi-objective problem (MOP).

In order to carry out the above transformation, the analyst has to choose between different stochastic efficiency concepts, three of which are described below. Details on some other efficiency concepts like Minimum Risk efficiency or Efficiency in Probabilities can be found in [8,16].

Each of these stochastic efficiency concepts is associated with a different DM risk attitude [16]

- *Expected Value efficiency*
  Under this concept of efficiency, the original SMOP is turned into the following multi-objective program:
  (2) $$\begin{array}{cc} opt_{x\in F} & \left({\bar{z}}_{1}\left(x\right),\cdots ,{\bar{z}}_{m}\left(x\right)\right), \end{array}$$
  where ${\bar{z}}_{k}\left(x\right)=E\left\{{\tilde{z}}_{k}\left(x,\tilde{\xi }\right)\right\}$ represents the expectation of the random variable defined by criterion *k*, for k=1,⋯,m.
  The Expected Value efficient set is denoted by $\varepsilon_{E}$.
  In this scenario, the DM does not show any concern for the potential variability of random criteria. Risk attitude could be considered as being neutral [8].
- *Minimum Variance and Minimum Standard Deviation efficiency*
  In this case, the original SMOP problem is transformed into the following multi-objective program:
  $$\begin{array}{cc} \min_{x\in F} & \left(\sigma_{1}^{2}\left(x\right),\cdots ,\sigma_{m}^{2}\left(x\right)\right), \end{array}$$
  where $\sigma_{k}^{2}\left(x\right)=V\left\{{\tilde{z}}_{k}\left(x,\tilde{\xi }\right)\right\}$ denotes the variance of the random variable defined by the criterion *k*.
  The Minimum Variance efficient set will be denoted by $\varepsilon_{\sigma^{2}}$. This efficient set does not change if we use the standard deviation $\sigma_{k}\left(x\right)$ of each random criterion in stead of the variance.
  (3) $$\begin{array}{cc} \min_{x\in F} & \left(\sigma_{1}\left(x\right),\cdots ,\sigma_{m}\left(x\right)\right). \end{array}$$
  So, we are talking indistinctly about Minimum Variance efficiency, and Minimum Standard Deviation efficiency $\varepsilon_{\sigma }$.
  In this approach, the risk attitude of the DM is interpreted as being extreme risk aversion [8] because the variance has been considered to be the main risk measure [24]. Risk averse DMs want to minimize variance even if they are solving a maximization problem.
- *Expected Value Standard Deviation efficiency*
  This concept can be interpreted as being a mixture of the two defined above. The SMOP is transformed into the following multi-objective program, doubling the original dimenison *m*. For *m* stochastic criteria in (1) we now have 2m criteria in the following MOP.
  (4) $$\begin{array}{cc} opt_{x\in F} & \left({\bar{z}}_{1}\left(x\right),\cdots ,{\bar{z}}_{m}\left(x\right),\sigma_{1}\left(x\right),\cdots ,\sigma_{m}\left(x\right)\right). \end{array}$$
  It is understood that, in this kind of formulation, the standard deviations are minimized, whereas the expected values can be maximized or minimized. The Expected Value Standard Deviation efficient set will be denoted by $\varepsilon_{E\sigma }$.
  The origin of this model can be found in Markowitz [24], in a portfolio selection scenario. This efficiency criterion can be assigned to a DM whose attitude towards risk is a balance between neutrality and extreme aversion attitudes. We call that a risk-moderate attitude.

From a technical point of view, the above three stochastic efficiency concepts are connected by using the notion of efficient set (A good exposition and definitions of efficiency, weak efficiency and associated sets can be found in chapter 2 in Ehrgott [25]). Developing the results in [16] for a general SMOP scenario, under general conditions (Strict convexity of the feasible set), the following relationship holds,

(5) $$\varepsilon_{E}\cup \varepsilon_{\sigma }\subseteq \varepsilon_{E\sigma }.$$

This relationship is going to be a cornerstone for this work. It ensures that efficient solutions to the Expected Value or Minimum Variance problems lie in the Expected Value Standard Deviation Efficient Set. In terms of risk aversion, solutions which are efficient for a risk-neutral or risk-averse DM, are also efficient for a risk-moderate DM. But there may be solutions that are suitable for a risk-moderate DM and not for the extremes.

Now we illustrate this relationship with an example.

**Example** **1.**  *Consider a SMOP with two decision variables, $x=\left(x_{1},x_{2}\right)\in R^{2}$ and two stochastic criteria:*

(6) $$\begin{array}{cc} \min_{x\in F} & ({\tilde{z}}_{1}\left(x_{1},x_{2}\right),{\tilde{z}}_{2}\left(x_{1},x_{2}\right)), \end{array}$$

*where*

$$\begin{array}{c} {\tilde{z}}_{1}\left(x_{1},x_{2}\right)=-7x_{1}-12x_{2}+\left(6x_{1}+5x_{2}\right){\tilde{\eta }}_{1}\\ {\tilde{z}}_{2}\left(x_{1},x_{2}\right)=-7x_{1}-16x_{2}+\left(4x_{1}+8x_{2}\right){\tilde{\eta }}_{2}, \end{array}$$

*which depend on the Normal $N(\mu ,\sigma^{2})$ and Poisson P(λ) random variables, ${\tilde{\eta }}_{1}∼N\left(1,4\right)$ and ${\tilde{\eta }}_{2}∼P\left(2\right)$.*

**Figure 1:**
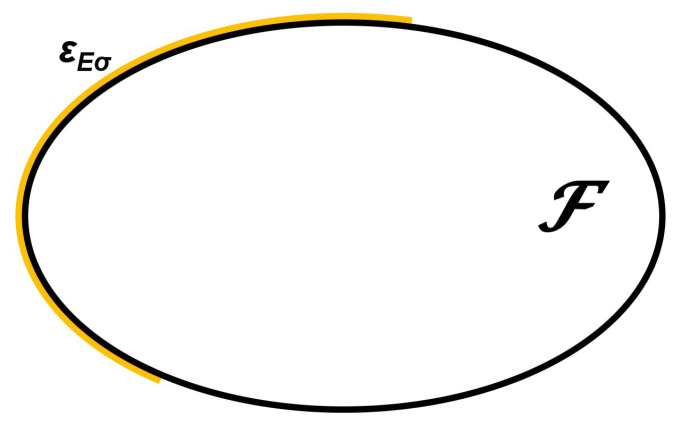
Feasible set in Example 1 and Expected Value standard Deviation Efficient Set.

*The feasible set is compact and strictly convex, $F=\{x\in R^{2}:\frac{{\left(x_{1}-2\right)}^{2}}{2}+\frac{{\left(x_{2}-2\right)}^{2}}{1}\leq 1\}$. i.e., a closed ellipse, which corresponds to the black line in Figure 1.*

A risk-moderate DM (not neutral, not averse) will find Expected Value Standard Deviation Efficient solutions,

$$\begin{array}{cc} \min_{x\in F} & ({\bar{z}}_{1}\left(x\right),{\bar{z}}_{2}\left(x\right),\sigma_{1}\left(x\right),\sigma_{2}\left(x\right)), \end{array}$$

these, in our case, being ${\bar{z}}_{1}\left(x\right)=-x_{1}-7x_{2}$, ${\bar{z}}_{2}\left(x\right)=x_{1}$, $\sigma_{1}\left(x\right)=12x_{1}+10x_{2}$ and $\sigma_{2}\left(x\right)=\sqrt{2}\left(4x_{1}+8x_{2}\right)$. The efficient set $\epsilon_{E\sigma }$ for a risk-moderate DM is the yellow line in Figure 1.

A risk-neutral DM will find solutions using the MOP,

$$\begin{array}{cc} \min_{x\in F} & ({\bar{z}}_{1}\left(x\right),{\bar{z}}_{2}\left(x\right)), \end{array}$$

and on the other hand, an extreme risk-averse DM should find solutions for MOP,

$$\begin{array}{cc} \min_{x\in F} & (\sigma_{1}\left(x\right),\sigma_{2}\left(x\right)). \end{array}$$

**Figure 2:**
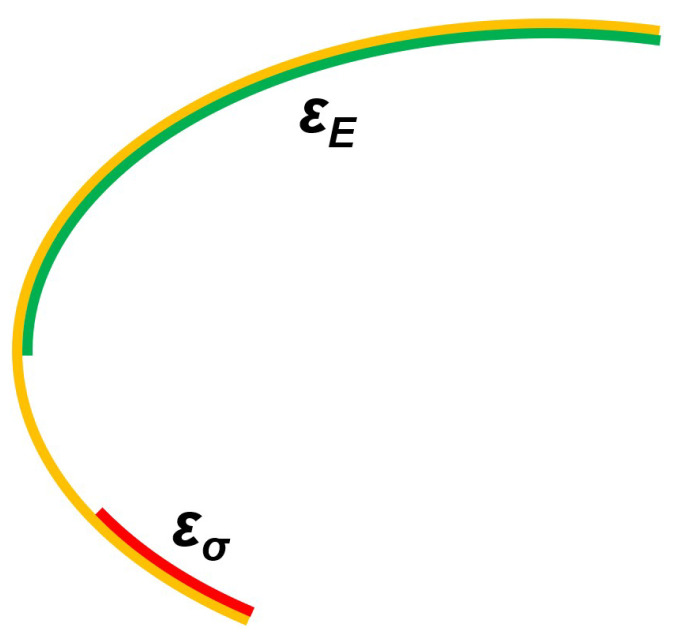
Efficient sets for a risk-neutral DM (green) and risk-averse DM (red).

The respective efficient sets, $\varepsilon_{E}$ and $\varepsilon_{\sigma }$ can be seen in Figure 2. The Expected Value efficient set is the green line, that is appropriate for a risk-neutral DM. The Minimum variance efficient set, suitable for a risk-averse DM, is the red line. It can be seen that both lie in $\epsilon_{E\sigma }$, yellow in Figure 2.

As expected from the relations in Equation (5), the relationship $\varepsilon_{E}\cup \varepsilon_{\sigma }\subset \varepsilon_{E\sigma }$ has been verified.

In this example we find an empty intersection so that we see a partition of $\varepsilon_{E\sigma }$. If we denote the complement of $\varepsilon_{E}$ as $\bar{\varepsilon_{E}}$ then the part of $\varepsilon_{E\sigma }$ which is not in either $\varepsilon_{E}$ or $\varepsilon_{\sigma }$ can be denoted as $\left(\bar{\varepsilon_{E}}\cap \bar{\varepsilon_{\sigma }}\right)\cap \varepsilon_{E\sigma }$. Then, using notation just said, $\left\{\varepsilon_{E},\varepsilon_{\sigma },\left(\bar{\varepsilon_{E}}\cap \bar{\varepsilon_{\sigma }}\right)\cap \varepsilon_{E\sigma }\right\}$ is a partition of $\varepsilon_{E\sigma }$.

In terms of preferences, the solutions in $\left(\bar{\varepsilon_{E}}\cap \bar{\varepsilon_{\sigma }}\right)\cap \varepsilon_{E\sigma }$, the part of the yellow line on Figure 2 which is not red or green, are suitable for a risk-moderate DM but not for an extreme risk-averse, or a risk-neutral, decision maker.

Nevertheless this does not happen in all cases. Examples are often found, in which the complement of $\varepsilon_{E}\cup \varepsilon_{\sigma }$ on $\varepsilon_{E\sigma }$ is empty.

## 3. Compromise Efficiency Concept in SMOP

As we have said, sometimes the analyst has to choose between different stochastic efficiency concepts as part of the process to solve the general SMOP using the multi-objective approach. These concepts are related with DM risk attitudes. In some cases, this choice appears as a conflict between two poles of the DM risk attitude: a neutral risk attitude, more focused on expected consequences, and the extreme averse risk attitude, more concerned with inherent uncertainty.

For solving this conflict, we propose a new stochastic efficiency concept that attempts to find a middle ground between these two poles.

Consider a SMOP as in Equation (1) where we want to minimize criteria. For each stochastic criterion ${\tilde{z}}_{k}\left(x,\tilde{\xi }\right)$, k=1,⋯,m, we define a parameter $\lambda^{k}\in \left[0,1\right]$ that represents the trade-off between the two extreme risk attitudes. If DM is risk-neutral for a criterion *k*, then the analyst assigns the value $\lambda^{k}=0$. If DM is risk-averse then the analyst assigns the value $\lambda^{k}=1$.

With this new parameters, we can define a new efficiency concept that extends those appeared in Section 2. In fact this new efficiency concept will be related with the Expected Value Standard Deviation efficiency concept. One of the differences between them is the dimension of the associated MOP, which will be equal to the original dimension of the SMOP, *m*.

IV. *Compromise Efficiency concept*
  Under this concept of efficiency, Equation (1), the original SMOP is turned into the following multi-objective program:
  (7) $$\begin{array}{cc} \min_{x\in F} & \left[\left(1-\lambda^{1}\right){\bar{z}}_{1}\left(x\right)+\lambda^{1}\sigma_{1}\left(x\right),\cdots ,\left(1-\lambda^{m}\right){\bar{z}}_{m}\left(x\right)+\lambda^{m}\sigma_{m}\left(x\right)\right]. \end{array}$$
  This program allows the implementation of the DMs attitude regarding risk aversion in each criterion, by changing the values of the $\lambda^{k}$ coefficients.
  Parameters $\lambda^{k}$, k=1,⋯,m, have a preferential interpretation in their range $\lambda^{k}\in \left[0,1\right]$. As we increase values of all the $\lambda^{k}$ we move from a risk neutral attitude towards risk aversion.
  For $\left(\lambda^{1},\cdots ,\lambda^{m}\right)=\left(0,\cdots ,0\right)$ we obtain efficient solutions for a risk-neutral DM, and for $\left(\lambda^{1},\cdots ,\lambda^{m}\right)=\left(1,\cdots ,1\right)$ solutions that are adequate for a risk-averse DM. Any other value of the vector $\left(\lambda^{1},\cdots ,\lambda^{m}\right)$ will provide efficient solutions for a risk-moderate DM.
  The closer the parameters are to 0, the closer the solution will be suitable for a risk-neutral DM, and, as we increase the values, we move towards risk aversion.
  The efficient set of program on Equation (7) will be denoted by $\varepsilon_{\left(\lambda^{1},\cdots ,\lambda^{m}\right)}$. When ∀k=1,⋯,m, $\lambda^{k}=0$ then the program in Equation (7) is identical to the one in Equation (2), so that
  (8) $$\varepsilon_{\left(0,\cdots ,0\right)}=\varepsilon_{E}\subset \varepsilon_{E\sigma }.$$
  When ∀k=1,⋯,m, $\lambda^{k}=1$ the program in Equation (7) is identical to the program in Equation (3), so
  (9) $$\varepsilon_{\left(1,\cdots ,1\right)}=\varepsilon_{\sigma }\subset \varepsilon_{E\sigma }.$$
  For intermediate values, ∀k=1,⋯,m, $\lambda^{k}\in \left[0,1\right]$, the corresponding efficient set $\varepsilon_{\left(\lambda^{1},\cdots ,\lambda^{m}\right)}$ is included in $\varepsilon_{E\sigma }$.
  Proof of this statement comes from the fact that when we find a efficient solution of MOP on Equation (7) particularized for a risk aversion coefficients vector $\left(\lambda^{1},\cdots ,\lambda^{m}\right)$ using the weighted sums method, the program we obtain is equal to one of the programs that we will obtain using the same method on MOP on Equation (4). So each efficient solution of (7) is also efficient for (4).
  Then, for every set of values of $\lambda^{k}\in \left[0,1\right]$, k=1,⋯,m, we have
  (10) $$\varepsilon_{\left(\lambda^{1},\cdots ,\lambda^{m}\right)}\subset \varepsilon_{E\sigma }.$$
  Sometimes the DM risk attitude does not require selecting a different degree of risk aversion for each criterion, thus in such cases all the trade-off parameters are equal,
  $$\lambda =\lambda^{1}=\cdots =\lambda^{m}.$$
  In this case the associated MOP is:
  (11) $$\begin{array}{cc} \min_{x\in F} & \left[\left(\left(1-\lambda \right){\bar{z}}_{1}\left(x\right)+\lambda \sigma_{1}\left(x\right)\right),\cdots ,\left(\left(1-\lambda \right){\bar{z}}_{m}\left(x\right)+\lambda \sigma_{m}\left(x\right)\right)\right], \end{array}$$
  This is a particular case of the Compromise Efficiency concept where the risk attitude is unique for all criteria. We denote the efficient set as $\varepsilon_{\lambda }$.

In Decision Analysis it is not enough to provide the whole efficient set corresponding to the DM’s attitude towards risk as a solution. The DM needs specific solutions, so we need to find a suitable one inside these efficient sets. On the other hand, we need that these solutions can be interpreted from a preferential point of view.

We may use different methods to find solutions for multicriteria programs [26]. We will consider four of them, but there are more. Selection of an specific method by the analyst shall depend on a new interaction with DM. We consider two methods under optimizing philosophy, which provide efficient solutions, and then a other two which produce solutions under a satisfizing approach.

Let us start with the weighted additive method, also known as weighted sums method (WS) [25]. If we consider importance weights $\left(\omega_{1},\cdots ,\omega_{m}\right)\in R_{+}^{m}$ then the weighted sums program is:

(12) $$\begin{array}{cc} \min_{x\in F} & \sum_{i=1}^{m}\left[\omega_{i}\left(1-\lambda^{i}\right){\bar{z}}_{i}\left(x\right)+\omega_{i}\lambda^{i}\sigma_{i}\left(x\right)\right]. \end{array}$$

Solutions to this program will be in $\varepsilon_{\left(\lambda^{1},\cdots ,\lambda^{m}\right)}$. Importance weights $\left(\omega_{1},\cdots ,\omega_{m}\right)$ admit [26] a preferential interpretation, relative importance of criteria, so shall be fixed by analyst in interaction with the DM.

Second method we consider is Compromise Programming (CP) [26,27]. This method produces solutions minimizing the distance to an ideal point, unreachable, where each criterion on a MOP would reach its optimum value if considered alone, without counting on other criteria. The program which provides compromise solutions to the MOP on Equation (7) is

(13) $$\min_{x\in X}{\left(\sum_{i=1}^{m}\omega_{i}^{p}{\left[\left(\left(1-\lambda^{i}\right){\bar{z}}_{i}\left(x\right)+\lambda^{i}\sigma_{i}\left(x\right)\right)-{\left(\left(1-\lambda^{i}\right){\bar{z}}_{i}+\lambda^{i}\sigma_{i}\right)}^{I}\right]}^{p}\right)}^{\frac{1}{p}}$$

for 1≤p<∞ and

$$\min_{x\in X}\max_{i=1,\cdots ,m}\omega_{i}\left[\left(\left(1-\lambda^{i}\right){\bar{z}}_{i}\left(x\right)+\lambda^{i}\sigma_{i}\left(x\right)\right)-{\left(\left(1-\lambda^{i}\right){\bar{z}}_{i}+\lambda^{i}\sigma_{i}\right)}^{I}\right]$$

for p=∞.

We have denoted the ideal value of each criterion at (7) as ${\left(\left(1-\lambda^{k}\right){\bar{z}}_{k}+\lambda^{k}\sigma_{i}\right)}^{I}$, k=1,⋯,m. Note that once the value of λ is fixed, each ideal value will be constant. CP general formulation includes importance weights $\left(\omega_{1},\cdots ,\omega_{m}\right)$ as in WS formulttion, and also DM rationality can be reflected in parameter p ∈[1,∞]. The closest to 1, solutions found will minimize the sum of discrepancies from ideal values, and the highest the value, solutions found will minimize the greatest discrepancy.

So, values of parameter p and $\left(\omega_{1},\cdots ,\omega_{m}\right)$ shall be determined by analyst by interacting with DM. CP allows finding efficient solutions as WS. The difference between the two is that allows also reflecting rationality of DM wrt to allowed discrepancies from an ideal solution.

Under a satisfizing approach, we consider the Weighted Goal Programming formulation [26,28,29,30] (WGP). On this method, besides parameter λ, rationality of DM is shown on aspiration levels for criteria. Consider that, in a new interaction with the DM, the analyst fixes aspiration levels for expectations, ${\bar{z}}_{k}^{t}$ and for standard deviations $\sigma_{k}^{t}$, in both cases being k=1,⋯,m. Then we may deploy the MOP in Equation (7),

(14) $$\begin{array}{ccc} \min & \sum_{i=1}^{m}p_{i} & \\ s.t: & \left(1-\lambda^{k}\right){\bar{z}}_{k}\left(x\right)+\lambda^{k}\sigma_{k}\left(x\right)+n_{k}^{E}-p_{k}^{E}=\left(1-\lambda^{k}\right){\bar{z}}_{k}^{t}+\lambda^{k}\sigma_{k}^{t}, & k=1,\cdots ,m\\ \; & p_{k},n_{k},\geq 0, & k=1,\cdots ,m.\\ \; & x\in F \end{array}$$

DM preferences are included in the model by parameters λ, ${\bar{z}}_{k}^{t}$ and ${\bar{z}}_{k}^{t}$ on the soft restrictions.

Solutions to MOP in Equation (14) may not be efficient [31] if aspiration levels are reachable. To obtain efficient satisfizing solutions we may use [30] the straight restoration method which for this case leads to Equation (15). If, on solving program (14) deviation variables reach values $p_{i}^{*}$, k=1,⋯,m, then we shall have to solve a second program:

(15) $$\begin{array}{ccc} \max & \sum_{i=1}^{m}n_{i} & \\ s.t: & \left(1-\lambda^{k}\right){\bar{z}}_{k}\left(x\right)+\lambda^{k}\sigma_{k}\left(x\right)+n_{k}^{E}-p_{k}^{E})=\left(1-\lambda^{k}\right){\bar{z}}_{k}^{t}+\lambda^{k}\sigma_{k}^{t}, & k=1,\cdots ,m\\ \; & p_{k}=p_{k}^{*} & k=1,\cdots ,m,\\ \; & n_{k}\geq 0, & k=1,\cdots ,m,\\ \; & x\in F. \end{array}$$

This program will provide solutions which, keeping the degree of accomplishment of goals achieved by the solutions in program (14), will also be efficient.

Finally, we propose an alternative WGP approach, dealing with expectations and standard deviations separately, including the trade off parameter on the achievement function. That is, we propose to find solutions to (7) solving the MOP:

(16) $$\begin{array}{ccc} \min & \sum_{i=1}^{m}\left[\left(1-\lambda^{i}\right)p_{i}^{E}+\lambda^{i}p_{i}^{\sigma }\right] & \\ s.t: & {\bar{z}}_{k}\left(x\right)+n_{k}^{E}-p_{k}^{E}={\bar{z}}_{k}^{t}, & k=1,\cdots ,m,\\ \; & \sigma_{k}\left(x\right)+n_{k}^{\sigma }-p_{k}^{\sigma }=\sigma_{k}^{t}, & k=1,\cdots ,m,\\ \; & p_{k}^{E},n_{k}^{E},p_{k}^{\sigma },n_{k}^{\sigma }\geq 0, & k=k=1,\cdots ,m,\\ \; & x\in F. \end{array}$$

DM preferences on each criterion are included in the program by parameters $\lambda^{k}$, ${\bar{z}}_{k}^{t}$ and ${\bar{z}}_{k}^{t}$, k=1,⋯,m. As in the previous case, WGP, when aspiration levels fixed are reachable, we need to apply the straight restoration procedure to obtain efficient solutions.

Let us consider a practical case.

## 4. Case Study

Consider a Decision Maker, Health Authority, who, in order to rule preventive measures to mitigate the propagation of a pandemic, needs to fix the values of two variables, $x=(x_{1},x_{2})$. Say $x_{1}$ represents the time measures are going to be in force, and $x_{2}$ their intensity.

Decision has to be taken considering two criteria, ${\tilde{z}}_{1}\left(x\right)$ is a measure of the propagation of the pandemic, and ${\tilde{z}}_{2}\left(x\right)$ a measure of unwanted side effects on other aspects of health. Both have to be minimized, and are expressed on the same units.

Expressions for the criteria and the feasible set are the same as in Example 1. Thus, the expression of the decision problem is as in Equation (6).

$$\begin{array}{cc} \min_{x\in F} & ({\tilde{z}}_{1}\left(x_{1},x_{2}\right),{\tilde{z}}_{2}\left(x_{1},x_{2}\right)), \end{array}$$

where

$$\begin{array}{c} {\tilde{z}}_{1}\left(x_{1},x_{2}\right)=-7x_{1}-12x_{2}+\left(6x_{1}+5x_{2}\right){\tilde{\eta }}_{1}\\ {\tilde{z}}_{2}\left(x_{1},x_{2}\right)=-7x_{1}-16x_{2}+\left(4x_{1}+8x_{2}\right){\tilde{\eta }}_{2}, \end{array}$$

which depend on the Normal $N(\mu ,\sigma^{2})$ and Poisson P(λ) random variables, ${\tilde{\eta }}_{1}∼N\left(1,4\right)$ and ${\tilde{\eta }}_{2}∼P\left(2\right)$. The feasible set is a closed ellipse, $F=\{x\in R^{2}:\frac{{\left(x_{1}-2\right)}^{2}}{2}+\frac{{\left(x_{2}-2\right)}^{2}}{1}\leq 1\}$.

We are going to use the simplified expression of the Compromise Efficiency Concept, Equation (11), for m=2.

(17) $$\begin{array}{cc} \min_{x\in F} & \left[\left(1-\lambda \right){\bar{z}}_{1}\left(x\right)+\lambda \sigma_{1}\left(x\right),\left(1-\lambda \right){\bar{z}}_{2}\left(x\right)+\lambda \sigma_{2}\left(x\right)\right], \end{array}$$

Particularized for this SMOP is then

(18) $$\begin{array}{cc} \min_{x\in F} & \left[\left(1-\lambda \right)\left(-x_{1}-7x_{2}\right)+\lambda \left(12x_{1}+10x_{2}\right),\left(1-\lambda \right)x_{1}+\lambda \left(\sqrt{2}\left(4x_{1}+8x_{2}\right)\right)\right]. \end{array}$$

The analyst wants to provide solutions according to the DM’s attitude on risk aversion, represented by parameter λ, equal for all criteria. All importance weights have been considered to be equal throughout the practical case. Also we have used normalization factors on finding specific solutions, as is usual procedure in CP and GP. But they’re not explicitly shown in the programs to keep them simple.

Let us start with the weighted sums method considered in Equation (12). Particularized for this case is:

(19) $$\begin{array}{cc} \min_{x\in F} & \left[\left(1-\lambda \right)\left(-x_{1}-7x_{2}\right)+\lambda \left(12x_{1}+10x_{2}\right)+\left(1-\lambda \right)x_{1}+\lambda \sqrt{2}\left(4x_{1}+8x_{2}\right)\right]. \end{array}$$

**Figure 3:**
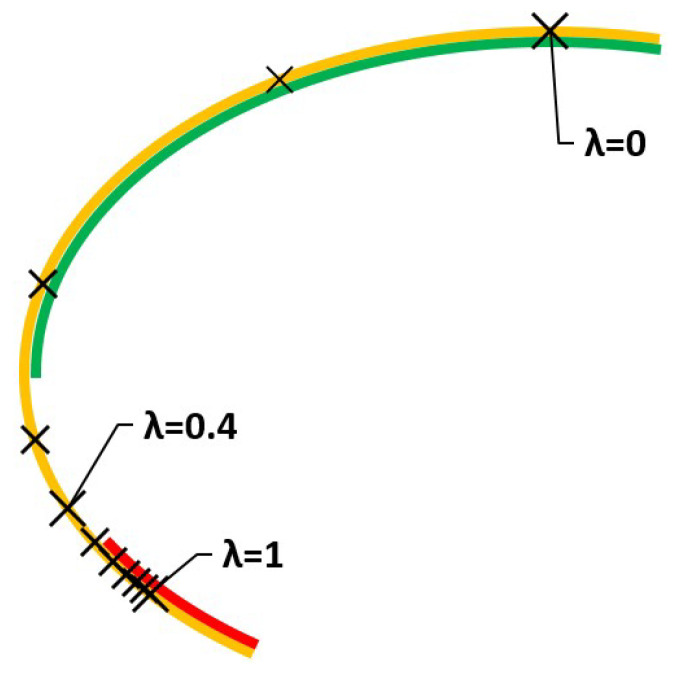
Solutions using the weighted sums method and efficient sets.

Solutions to this program will be in $\varepsilon_{\lambda }\subset \varepsilon_{E\sigma }$ and are shown on Table 1 and Figure 3 for different values of λ.

Solutions evolve as the value of λ is increased. For small values, there are suitable solutions for risk-neutral DMs, green line. As we increase the value we move towards solutions for a risk-averse DM, red line.

Suppose the DM, Health Authority, wants to obtain minimum values for both the criteria, but also wants to know what is going to happen. Let us say that the DM chooses λ=0.4, that is, closer to risk-neutral than to extreme risk-averse. The solution is shown in the graph in Figure 3.

Now we find solutions using CP, developing the program on Equation (13). Assume DM rationality leads to p =1. Ideal values for criteria depend on the value of λ, so we denote them shortly by $y{\left(\lambda \right)}_{k}^{I}$ for each criteria k=1,2. The particularized expression is then

(20) $$\min_{x\in X}\left[\left(\left(1-\lambda \right)\left(-x_{1}-7x_{2}\right)+\lambda \left(12x_{1}+10x_{2}\right)\right)-y{\left(\lambda \right)}_{1}^{I}+\left(\left(1-\lambda \right)x_{1}+\lambda \sqrt{2}\left(4x_{1}+8x_{2}\right)\right)-y{\left(\lambda \right)}_{2}^{I}\right]$$

**Figure 4:**
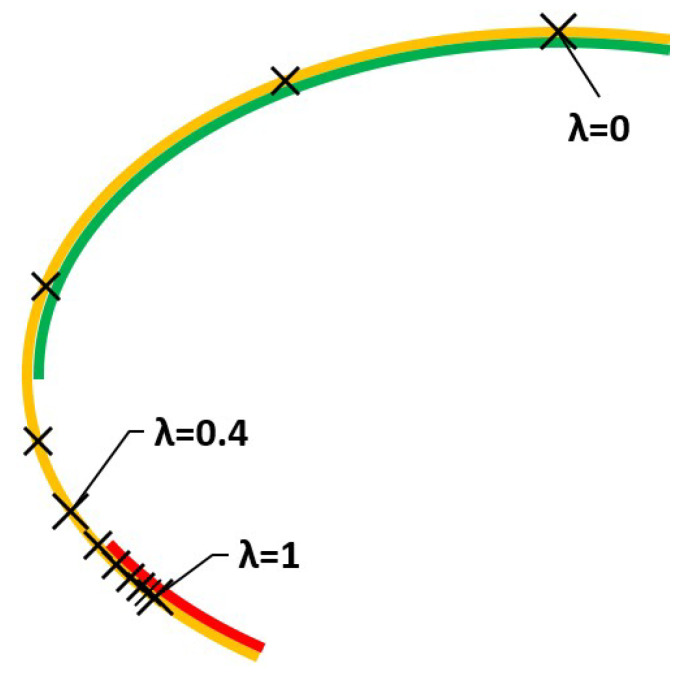
Solutions using compromise programming for *p* =1.

Solutions are in Figure 4. Note in this case that the movement is perfectly monotonous and smooth, comparable to those in Figure 3, obtained using the weighted sums method.

In order to use WGP formulation, besides parameter λ to reflect risk aversion, rationality of DM is shown on aspiration levels for criteria. Consider that, in a new interaction with the DM, the analyst fixes aspiration levels for expectations, ${\bar{z}}_{k}^{t}$ and for standard deviations $\sigma_{k}^{t}$, in both cases being k=1,2, as follows.

$$\begin{array}{ccc} {\bar{z}}_{1}^{t} & = & -16\\ {\bar{z}}_{2}^{t} & = & 2\\ \sigma_{1}^{t} & = & 44\\ \sigma_{1}^{t} & = & 34 \end{array}$$

Actual aspiration levels to be used are $\left(1-\lambda \right){\bar{z}}_{k}^{t}+\lambda \sigma_{k}^{t}$, k=1,2, since we have a two-dimension problem on Equation (17). MOP to solve using WGP is then

(21) $$\begin{array}{cc} \min & \left(p_{1}+p_{2}\right)\\ s.t: & \left(1-\lambda \right)\left(-x_{1}-7x_{2}\right)+\lambda \left(12x_{1}+10x_{2}\right)+n_{1}-p_{1}=-16\left(1-\lambda \right)+44\lambda ,\\ \; & \left(1-\lambda \right)x_{1}+\lambda \sqrt{2}\left(4x_{1}+8x_{2}\right)+n_{2}-p_{2}=2\left(1-\lambda \right)+34\lambda ,\\ \; & p_{1},n_{1},p_{2},n_{2}\geq 0,\\ \; & x\in F. \end{array}$$

Aspiration levels are reachable, so there is a nonempty set of satisficing solutions. So solutions to MOP in Equation (14) are sensitive to the seed used, and they may not be efficient [31]. To obtain efficient satisfizing solutions we may use [30] the straight restoration method which for this case leads to Equation (22).

(22) $$\begin{array}{cc} \max & \left(n_{1}+n_{2}\right)\\ s.t: & \left(1-\lambda \right)\left(-x_{1}-7x_{2}\right)+\lambda \left(12x_{1}+10x_{2}\right)+n_{1}-p_{1}=-16\left(1-\lambda \right)+44\lambda ,\\ \; & \left(1-\lambda \right)x_{1}+\lambda \sqrt{2}\left(4x_{1}+8x_{2}\right)+n_{2}-p_{2}=2\left(1-\lambda \right)+34\lambda ,\\ \; & p_{1}=p_{1}^{*},\\ \; & p_{2}=p_{2}^{*},\\ \; & n_{1},n_{2}\geq 0,\\ \; & x\in F. \end{array}$$

**Figure 5:**
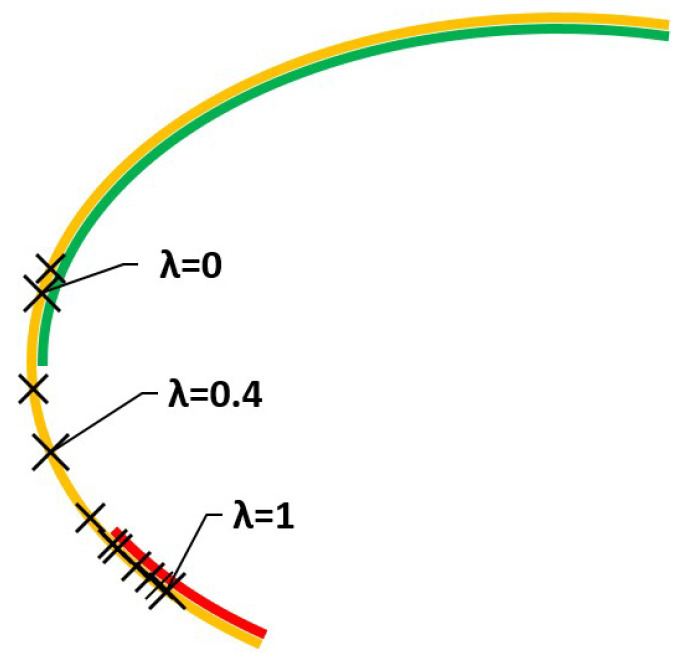
Solutions using WGP under the satisficing philosophy plus straight restoration.

We have used seed $\left(x_{1},x_{2}\right)=\left(4,4\right)$ on program in Equation (21) and used results on program on Equation (22). Running the two programs we have obtained the overall efficient solutions in Figure 5. Note that the monotonicity of solutions with respect to λ is not as clean as in the WS solutions in Figure 3.

Finally, we propose dealing with expectations and standard deviations separately on the achievement function, as in Equation (16). That is, we find solutions to (17) solving:

(23) $$\begin{array}{ccc} \min & \left[\left(1-\lambda \right)\left(p_{1}^{E}+p_{2}^{E}\right)+\lambda \left(p_{1}^{\sigma }+p_{2}^{\sigma }\right)\right] & \\ s.t: & \left({\bar{z}}_{1}\left(x\right)+n_{1}^{E}-p_{1}^{E}\right)=-x_{1}-7x_{2}, & \\ \; & \left({\bar{z}}_{2}\left(x\right)+n_{2}^{E}-p_{2}^{E}\right)=x_{1}, & \\ \; & \left(\sigma_{1}\left(x\right)+n_{1}^{\sigma }-p_{1}^{\sigma }\right)=12x_{1}+10x_{2}, & \\ \; & \left(\sigma_{2}\left(x\right)+n_{2}^{\sigma }-p_{2}^{\sigma }\right)=\sqrt{2}\left(4x_{1}+8x_{2}\right), & \\ \; & p_{k}^{E},n_{k}^{E},p_{k}^{\sigma },n_{k}^{\sigma }\geq 0, & k=1,2,\\ \; & x\in F. \end{array}$$

**Figure 6:**
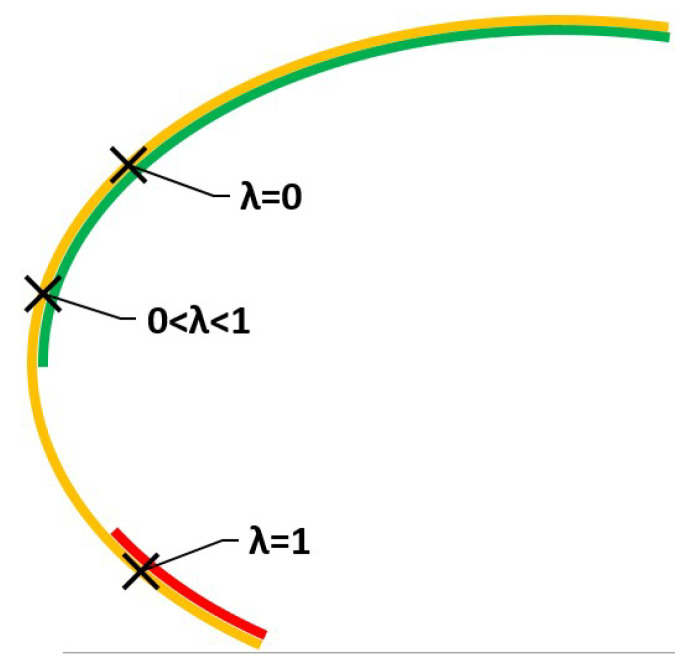
Solutions using WGP separately for expected values and standard deviations, under the satisficing philosophy plus straight restoration.

Like in the previous case we need to apply a straight restoration procedure to find efficient solutions. We can see solutions in Figure 6. In this case solutions for λ∈(0,1) are coincident, so there is not such as clear and neat movement as with other solving methods applied to the Compromise Efficiency concept.

## 5. Conclusions

We have proposed a model to help decision analysts when DM rationality is in a middle ground between extremes with respect to risk attitudes: the neutral risk attitude, more focused on expected consequences, and the extreme averse risk attitude, more concerned with inherent uncertainty.

The path between these two extreme risk attitudes is covered, for each criterion, by the values of a trade-off parameter λ∈[0,1].

This kind of proposal, reflecting the trade-off between two extreme situations using a continuous parameter, has already been made in other fields such as Social Choice, in which the analyst tries to consider simultaneously two desirable social properties in the analysis: the majority and the minority principles, see [32]. Or in Decision Theory where Hurwicz introduced his classical criterion with respect to optimism and pessimism [12].

In the newly proposed model, this λ-parameter can be interpreted as being the degree of risk to be assumed by the DM during the decision process. This perspective permits DM to avoid a possible dilemma in risk attitude, when the decision has to be viewed as strictly “one off”.

This degree of risk aversion can be uniform, the same for all criteria or can be individualized for different criteria. This fact permits our model to be more adaptive.

To obtain values for these parameters, the analyst will need to interact with the DM, or DMs in the case of a group decision-making problem. But this interaction is not difficult since DM will only need to choose a value between 0 and 1 fot the degree of risk aversion on each of the criteria in which the decision will be supported.This fact enables the DM not to forced to face the dilemma as a whole. And presents a much easier and straight procedure than the elicitation process of a multiattribute utility function.

As seen in the introduction, most Decision Analysis methods which consider risk, stochastic or fuzzy environments, do require the use of one utility function which, if the decision is taken considering several criteria, needs to be a multiattribute utility function. These are found using more or less complicated elicitation processes [9] which require making assumptions regarding the nature of such function (additive, multiplicative, etc.) which constrain the possibility of reflecting the real nature of potentially diverse Decision Makers.

On the new proposed model we do not need to consider an utility function since DM rationality is determined by separate parameters as said.

Actual outputs of the model are multicriteria optimization problems which define efficient sets. That is, sets of solutions who are considered to be Pareto efficient according to the rationality of the DM. This leaves analyst with flexibility to suggest specific solutions, using the resolution processes described at the end of Section 3 or others, based on other aspects of rationality.

Once solutions are proposed using this new model, it is easy to perform a sensitivity analysis by the analyst that would permit the DM to explore all the solutions by changing the values of the λ-parameters. This is the case when the risk attitude can be not stated a priori.

As seen in the introduction and in the case study, stochastic multicriteria decision situations arise on Health Care when dealing, as we can clearly see nowadays in the COVID-19 case, with measures to mitigate propagation of a pandemic. Are also common on practical Medicine when making diagnosis or prescribing medicines. Not to say in more general cases like environmental decisions, ecology, engineering, business management and classical fields such as portfolio selection for investments.

Investigation in this new model can be further developed. We have considered general random variables as criteria. A next step would be to see whether further results can be reached for the simple linear random and simple normal cases for which relationships with efficient sets for other efficiency concepts have been obtained at [16].

Also of interest in the future would be to consider a possible correlation between the random variables representing each criterion.

Further approaches could include the study of this situation from a Social Choice point of view, as in the current work we have considered risk and multicriteria.

It will also be useful to do research on the attitudes of a risk friendly (a gambler) DM, and see which solutions are suitable for this attitude.

Finally, we have not considered any normalization factors for the criteria, which are understood to be implemented in practice, but, due to the stochastic nature of the setting, may lead to new situations which could be worth investigating.

## Figures and Tables

**Table 1:** Solutions using the weighted sums method.

λ	$x_{1}$	$x_{2}$
0	2.0010	3.0000
0.1	1.2733	2.8579
0.2	0.6340	2.2588
0.3	0.6131	1.8045
0.4	0.7021	1.6029
0.5	0.7733	1.5024
0.6	0.8233	1.4453
0.7	0.8594	1.4088
0.8	0.8866	1.3834
0.9	0.9077	1.3648
1	0.9237	1.3513

## Data Availability

Not applicable.

## Abbreviations

The following abbreviations are used in this manuscript:

CP	Compromise Programming
DM	Decision Maker
GP	Goal Programming
MOP	Multicriteria Optimization Problem
SMOP	Stochastic Multicriteria Optimization Problem with Deterministic Constraints
WGP	Weighted Goal Programming
